# Supplementary material for: Electronic and Optical Properties of Two-Dimensional Tellurene: From First-Principles Calculations
Source: Nanomaterials (Basel). 2019 Jul 26;9(8):1075. doi: 10.3390/nano9081075 (PMC6722590; doi:10.3390/nano9081075)
Supplement: Supplementary file 1 [file nanomaterials-09-01075-s001.pdf]

nanomaterials

Supplementary Material for

Electronic and Optical Properties of Two-dimensional Tellurene: From First- Principles Calculations

David K. Sang †, Bo Wen †, Shan Gao, Yonghong Zeng, Fanxu Meng, Zhinan Guo * and Han Zhang *

Shenzhen Engineering Laboratory of Phosphorene and Optoelectronics, Collaborative Laboratory of 2D Materials for Optoelectronics Science and Technology, Engineering Technology Research Center for 2D Material Information Function Devices and Systems of Guangdong Province, Shenzhen University, Shenzhen 518060, China

* Correspondence: guozhinan@szu.edu.cn (Z.G.); hzhang@szu.edu.cn (H.Z.)

† These authors contributed equally to this work.

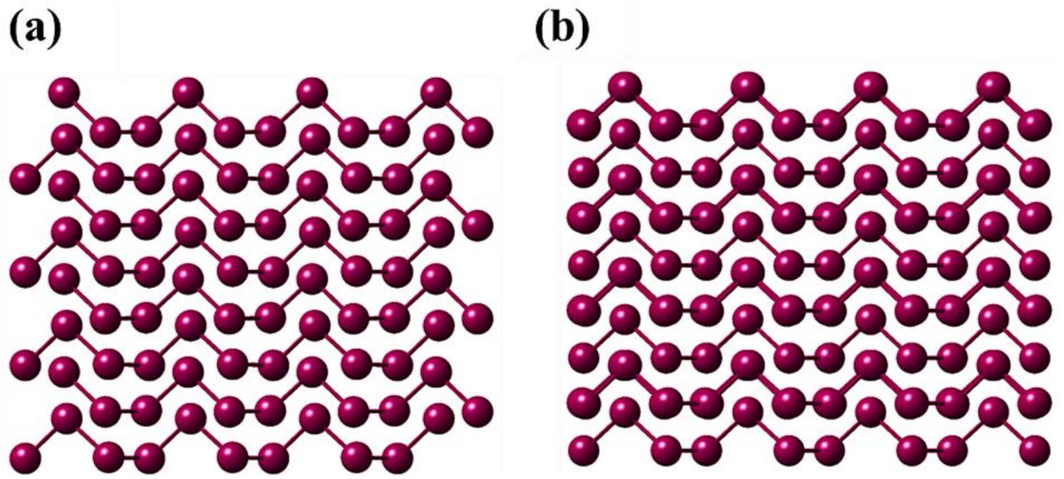

Figure S1. (**a**) Top view of even-layered number of 2D-Tellurene, (**b**) Top-view of odd-layered number of 2D-Tellurene.

Table Sl. Tabulation of valence band maximum (VBM) and conduction band minimum CBM energies values extracted from PBE-functional for monolayer (1 L) and few-layer (2 L–6 L) Tellurene.

**Number of Layer**	VBM (eV)	**CBM (eV)**
**1 L**	0.062	**0.904**
**2 L**	0.166	**0.687**
**3 L**	0.135	**0.498**
**4 L**	0.059	**0.437**
**5 L**	0.024	**0.372**
**6 L**	0.029	**0.312**

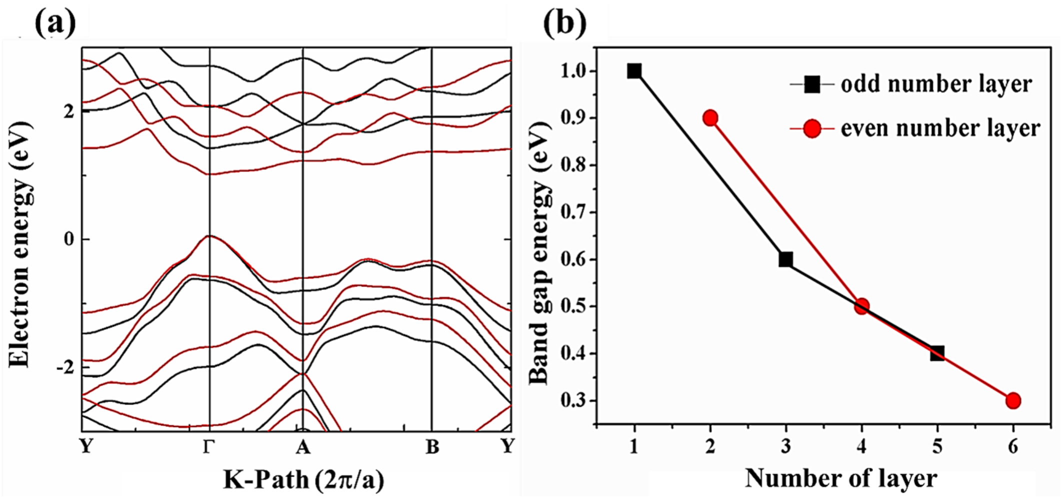

Figure S2. (**a**) Electronic band structure extracted from HSE06 (black) and DFT-PBE (red), (**b**) plot of band gap energy as a function of layer number showing odd- and even- number layer.

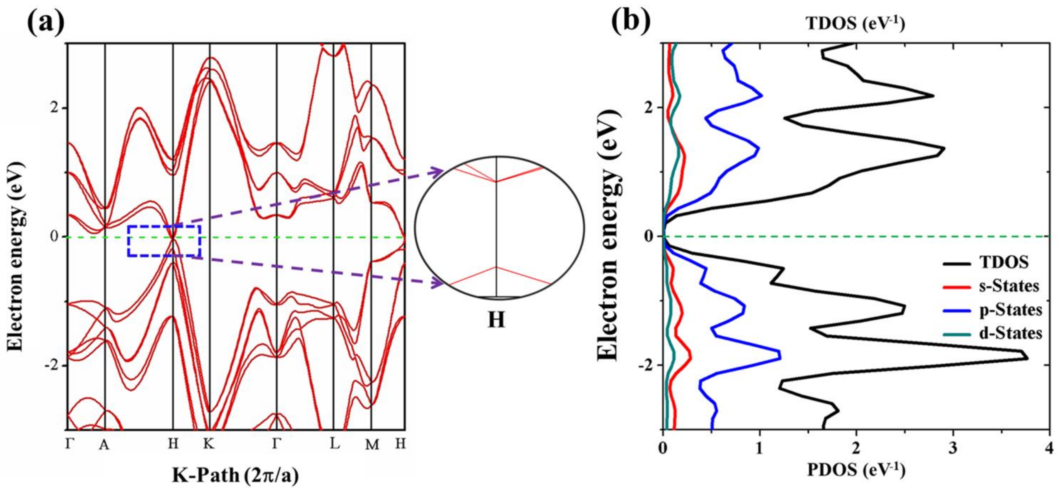

Figure S3. (**a**) Electronic band structure of bulk Tellurium (E_g = 0.32 eV), extracted from HSE06 functional, (**b**) Total density of state (TDOS) and Partial density of state (PDOS) of bulk Tellurium. The green dash line is Fermi energy level set to be 0 eV.

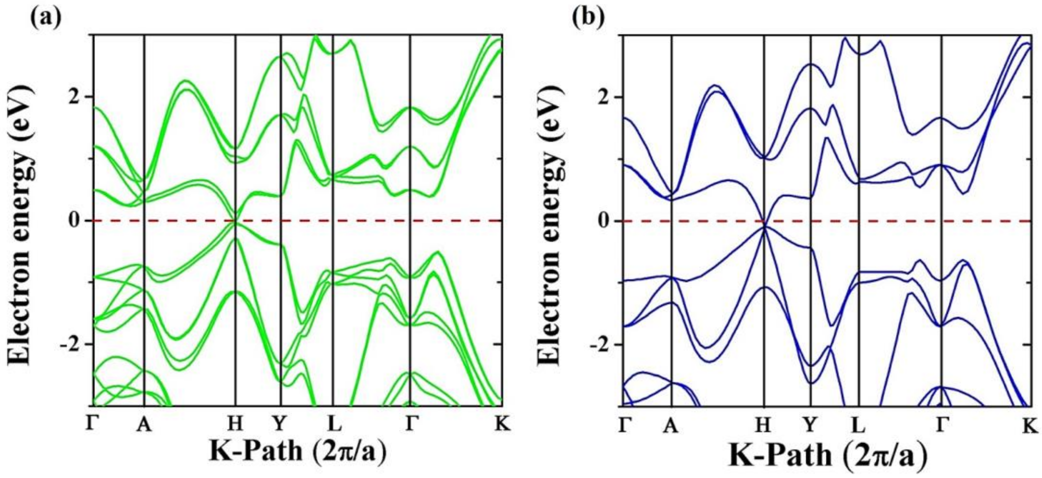

Figure S4. Electronic band structure of bulk Tellurium (**a**) with spin orbital coupling (SOC) (E_g = 0.052 eV), and (**b**) without SOC (E_g = 0.194 eV), extracted from GGA-PBE functional. The red dash line is Fermi energy level set to be 0 eV.

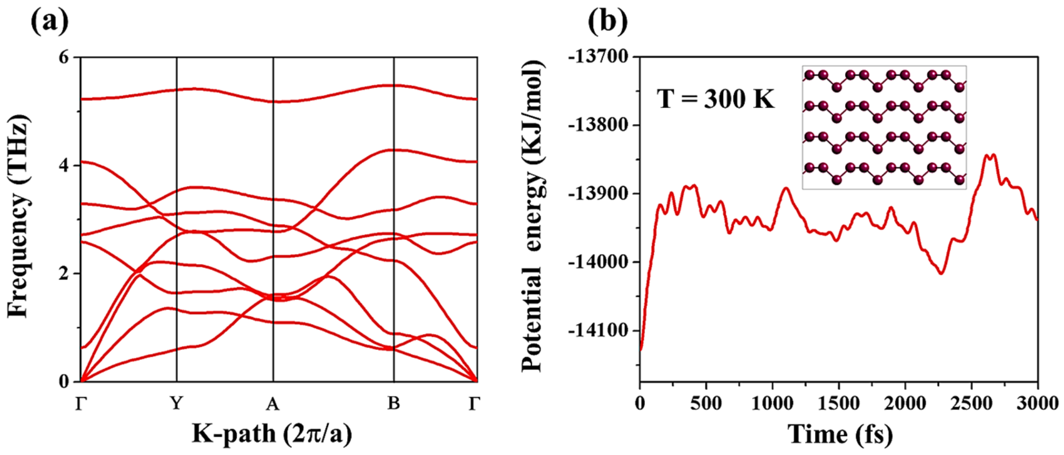

Figure S5. (**a**) Calculated phonon band dispersion of monolayer Tellurene, (**b**) Total potential energy fluctuation of monolayer Tellurene during AIMD simulations at the temperature of 300 K. The inset shows the snapshot of atomic configuration from the top view of monolayer Tellurene at the end of AIMD simulations.

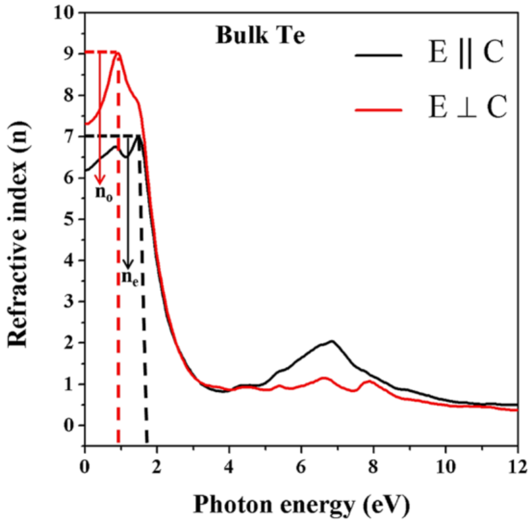

Figure S6. Calculated refractive index a long E ⊥ C (n_o = 9.06) and E || C (n_e = 7.00) polarization directions of the bulk Tellurium crystal.
